# A case of bilateral pachychoroid disease: polypoidal choroidal vasculopathy in one eye and peripheral exudative hemorrhagic chorioretinopathy in contralateral eye

**DOI:** 10.1186/s12886-021-02067-2

**Published:** 2021-09-04

**Authors:** Yorihisa Kitagawa, Hiroyuki Shimada, Akiyuki Kawamura, Koji Tanaka, Ryusaburo Mori, Hajime Onoe, Hiroyuki Nakashizuka

**Affiliations:** Department of Ophthalmology, School of Medicine, Nihon University, 1–6 Surugadai, Kanda, Chiyodaku, 101–8309 Tokyo, Japan

**Keywords:** Central serous chorioretinopathy, Pachychoroid disease, Peripheral exudative hemorrhagic choroidal retinopathy, Polypoidal choroidal vasculopathy, Punctate hyperfluorescent spot

## Abstract

**Background:**

We report a case of bilateral pachychoroid disease manifesting polypoidal choroidal vasculopathy (PCV) with punctate hyperfluorescent spot (PHS) in one eye, and peripheral exudative hemorrhagic choroidal retinopathy (PEHCR) with central serous chorioretinopathy (CSC) and PHS in the contralateral eye.

**Case presentation:**

: A 51-year-old healthy woman presented with complaint of blurred vision in her right eye. Corrected visual acuity was 20/20 in the right and 24/20 in the left eye. Fundus examination was normal in the left eye. In the right eye, fundus finding of an orange-red nodular lesion and optical coherence tomography (OCT) finding of polypoidal lesions led to a diagnosis of PCV. Four aflibercept intravitreal injections were performed in her right eye. After treatment, indocyanine green angiography (ICGA) confirmed residual polypoidal lesions with branching vascular networks and PHS with choroidal vascular hyperpermeability. OCT showed PHS associated with small sharp-peaked retinal pigment epithelium (RPE) elevation in peripheral fundus and small RPE elevation in posterior fundus. Based on the above findings, PCV with PHS was finally diagnosed in the right eye. Posttreatment corrected visual acuity in the right eye was 20/20. She presented again 32 months later, with complaint of vision loss in her left eye. Left corrected visual acuity was 20/20, and fundus examination showed mild vitreous hemorrhage. Vitrectomy was performed. In temporal midperipheral fundus, fluorescein angiography revealed CSC, and OCT showed pachychoroid. ICGA depicted abnormal choroidal networks and PHS in peripheral fundus. Furthermore, polypoidal lesions were confirmed by OCT. Based on the above findings, PEHCR and CSC with PHS was finally diagnosed in the left eye. Postoperative corrected visual acuity in the left eye was 20/20, and aflibercept intravitreal injection was performed for prevention of recurrence of vitreous hemorrhage.

**Conclusions:**

This is the first case report of PCV with PHS in one eye, and PEHCR with CSC and PHS in the contralateral eye. This case suggests that PCV, PEHCR, and CSC may be linked pathologies of pachychoroid spectrum disease.

## Background

Initially described by Yannuzzi in 1990, polypoidal choroidal vasculopathy (PCV) is a subtype of neovascular age‑related macular degeneration (AMD) commonly seen in the Asian population. PCV is usually located at the posterior pole in the macular or peripapillary region. It is a distinct abnormality of the choroidal vasculature with characteristic branching networks of choroidal vessels and surrounding polypoidal dilatation of the vessels [1, 2]. According to Japanese Study Group Guidelines, the diagnostic criteria for definite PCV are elevated orange-red lesions on fundus examination and/or polypoidal lesions on indocyanine green angiography (ICGA), and the criteria for probable PCV are only abnormal branching vascular network seen on ICGA or recurrent hemorrhagic or serous pigment epithelium detachment (PED) or both, without features of definite PCV [2]. A recent study has reported that that if OCT findings including sharp PED, PED notch, and double-layer sign are confirmed, PCV can be diagnosed clinically without ICGA [3].

Peripheral exudative hemorrhagic chorioretinopathy (PEHCR), which was named by Annesley in 1980, is typically found in older people, and peripheral mass lesions are often highly exudative and hemorrhagic. PEHCR is usually associated with PED, sometimes extending to the macula. On ICGA, polyp-like choroidal telangiectases and abnormal choroidal vascular networks are observed [4, 5].

While AMD (both early and exudative) is associated with choroidal thinning, PCV and central serous chorioretinopathy (CSC) are associated with pachychoroid. PEHCR is considered to be included in the pachychoroid disease spectrum due to the gradual increase in choroidal thickness from nasal towards temporal periphery, and the presence of pachyvessels [6]. Although there are many reports on PCV or PEHCR individually, the association between the two diseases has not been documented. We report a case of PCV with punctate hyperfluorescent spot (PHS) in one eye, and PEHCR with CSC and PHS in the contralateral eye, demonstrating the linkage of these conditions in pachychoroid spectrum disease.

## Case presentation

Written informed consent for publishing the clinical data and images was obtained from the patient. This case study was conducted in accordance with the tenets of the Declaration of Helsinki.

**Fig. 1:**
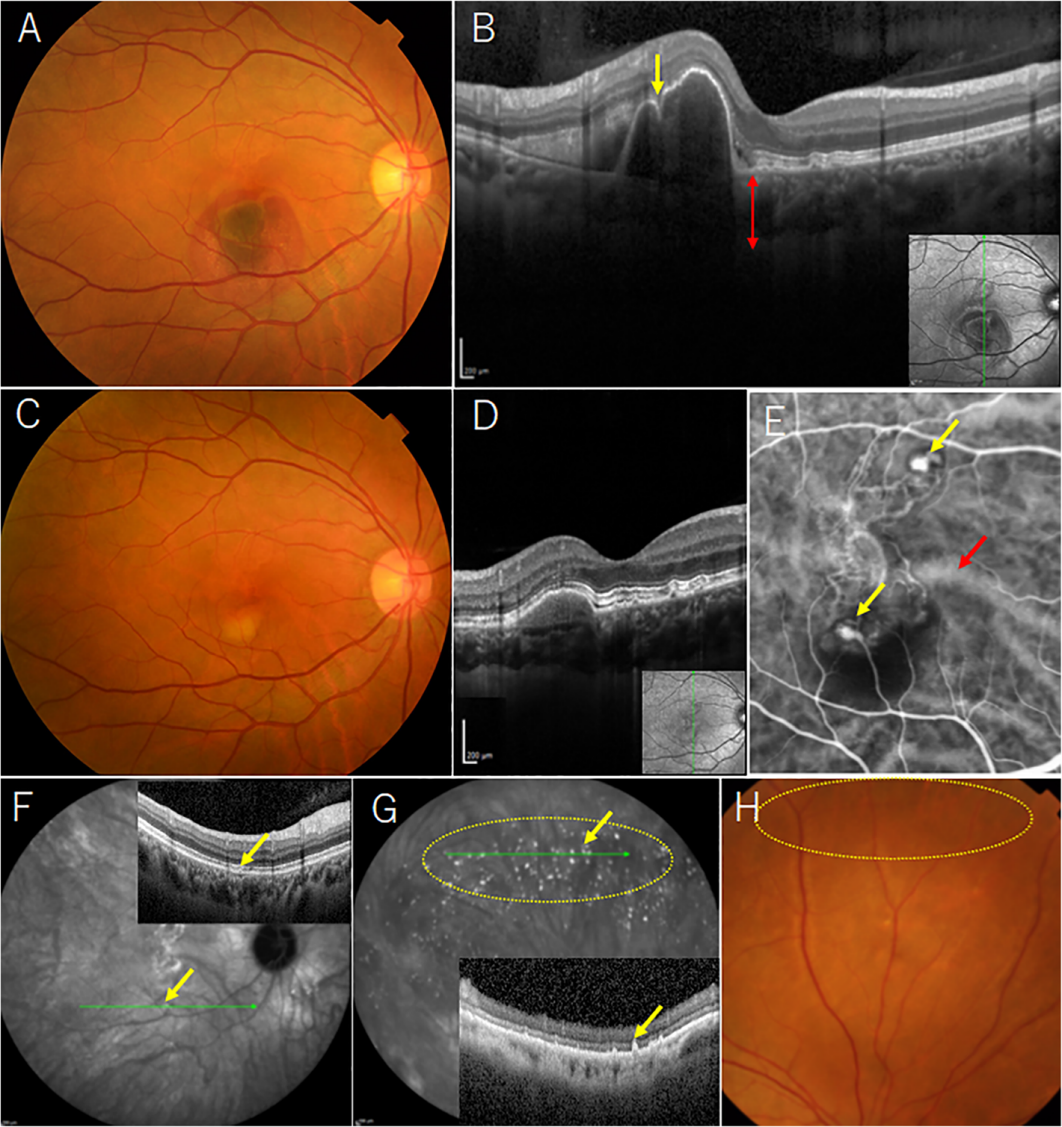
Image findings of the right eye before (**a, b**) and after four aflibercept intravitreal injections (**c-g**). **a** Fundus photograph shows an orange-red nodular lesion and hemorrhagic PED surrounded by subretinal hemorrhage in the macula. **b** OCT image shows tomographic notch sign (yellow arrow) and pachychoroid (red up-down arrow: 309 μm). **c** Subretinal hemorrhage is resolved and polypoidal lesions have improved. **d** OCT image shows disappearance of polypoidal lesion. **e** In early-phase ICGA image, polypoidal lesions (yellow arrows) are still present. Engorgement of choroidal vessels was found (red arrow). **f** Late-phase ICGA image depicts PHS (yellow arrow) in posterior fundus. OCT image at the site of PHS shows small RPE elevation (yellow arrow). **g** Late-phase ICGA image reveals PHS in peripheral fundus (yellow oval). OCT at this site shows small sharp-peaked RPE elevations (yellow arrow). **h** Fundus photograph shows no abnormality in the area where ICGA depicts PHS (yellow oval). PED: pigment epithelial detachment, OCT: optical coherence tomography, ICGA: indocyanine green angiography, PHS: punctate hyperfluorescent spots, RPE: retinal pigmented epithelium

A 51-year-old healthy woman presented with complaint of blurred vision in her right eye. Corrected visual acuity was 20/20 in the right and 24/20 in the left. No abnormalities in the fundus were observed in the left eye. In the right eye, fundus examination revealed an orange-red nodular lesion and hemorrhagic PED surrounded by subretinal hemorrhage in the macula region, leading to a diagnosis of PCV (Fig. 1a). OCT showed tomographic notch sign and pachychoroid (choroidal thickness 309 μm) (Fig. 1b). Four aflibercept intravitreal injections were performed in her right eye to resolve the lesions. After the intravitreal injections, subretinal hemorrhage was resolved and polypoidal lesions improved (Fig. 1c). OCT showed disappearance of polypoidal lesions (Fig. 1d). Early-phase ICGA showed residual polypoidal lesions and branching vascular networks in the macula. Engorgement of choroidal vessels was found (Fig. 1e). In posterior fundus, late-phase ICGA showed PHS apparently located at the center of the choroidal vascular hyperpermeability area, and OCT at the site with PHS showed small retinal pigment epithelium (RPE) elevation (Fig. 1f). In peripheral fundus, late-phase ICGA depicted PHS but no choroidal vascular hyperpermeability, and OCT of the area with PHS showed small sharp-peaked RPE elevations (Fig. 1 g). Fundus examination of the peripheral fundus showed no abnormality in the area where PHS was seen on ICGC (Fig. 1 h). Based on the above findings, PCV with PHS was finally diagnosed in the right eye. Postoperative corrected visual acuity in the right eye was 20/20.

**Fig. 2:**
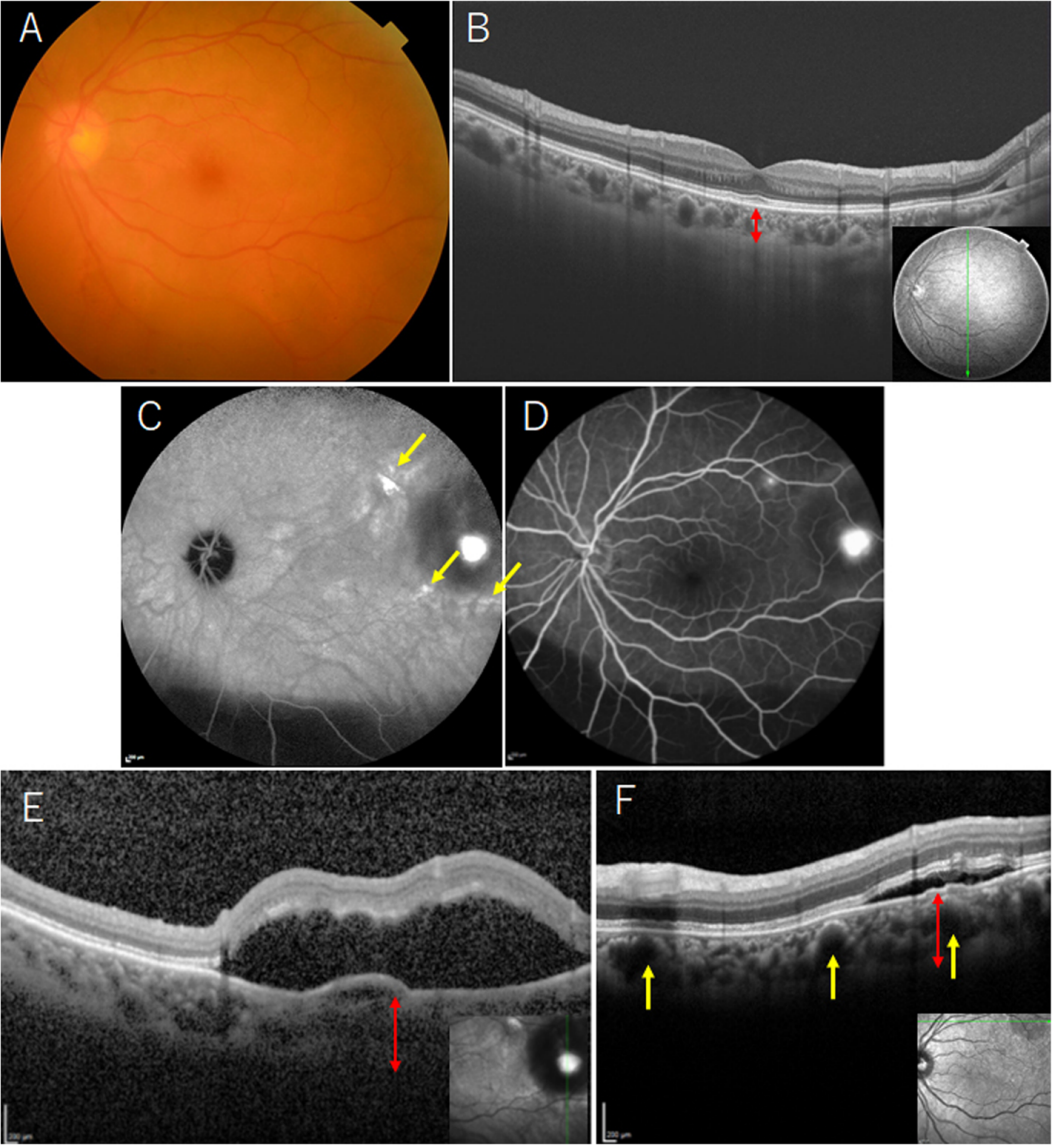
Fundus findings of the left eye after vitreous surgery. **a** Fundus photograph shows no abnormalities in posterior pole. **b** OCT image shows normal choroid thickness (red up-down arrow: 203 μm) in the macula and subretinal hemorrhage in inferior peripheral fundus. **c** Late-phase ICGA image shows choroidal vascular hyperpermeability with PHS (yellow arrows). **d** Fluorescein angiography shows two inkblot leaks. **e** In the inferior CSC lesion, pachychoroid (red up-down arrow: 419 μm) is observed. **f** In superior CSC lesion, pachychoroid (red up-down arrow: 439 μm) with pachyvessels (yellow arrows) are depicted. CSC: central serous chorioretinopathy, OCT: optical coherence tomography, ICGA: indocyanine green angiography

**Fig. 3:**
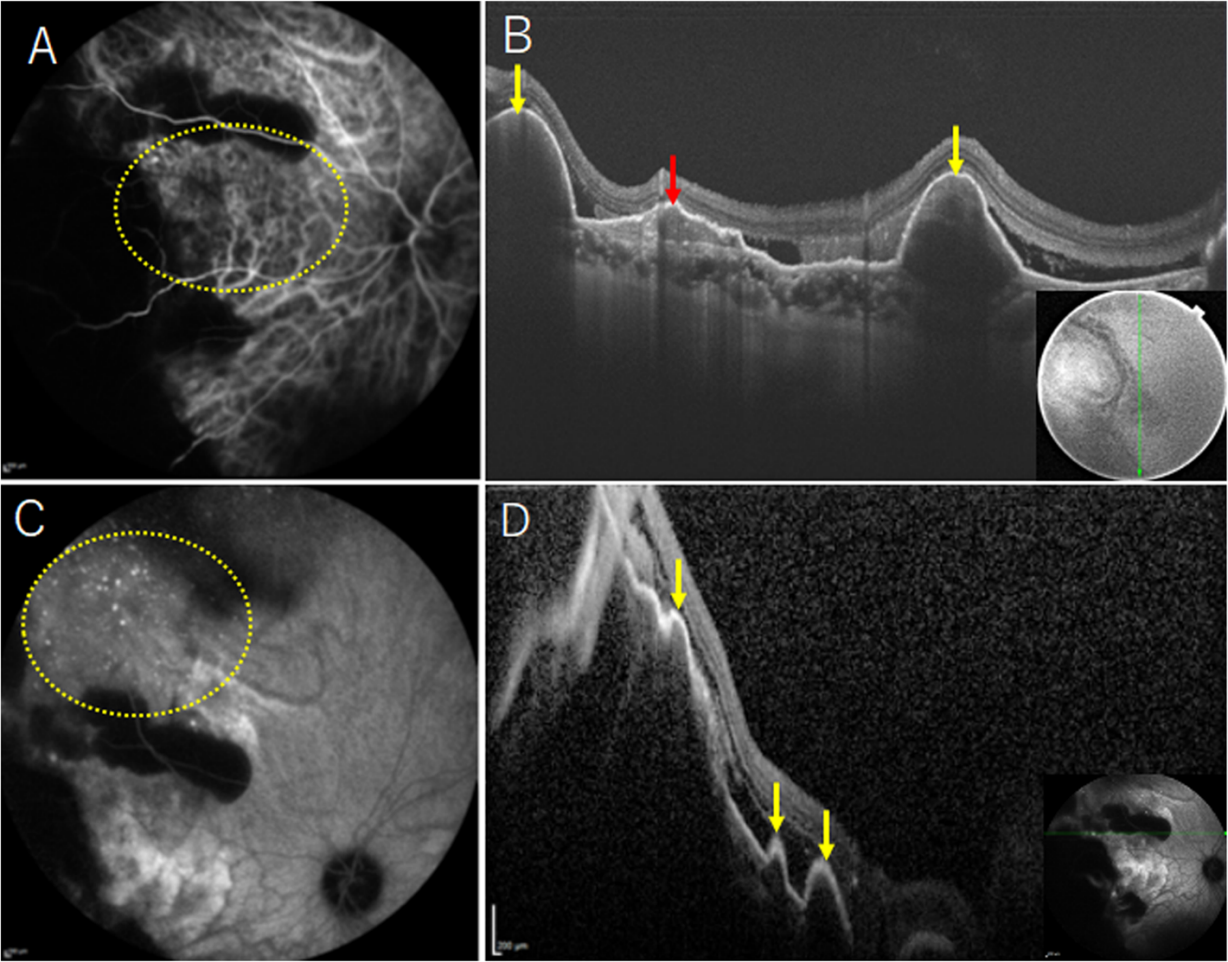
Peripheral retinal findings in the left eye after vitreous surgery. **a** ICGA reveals abnormal choroidal network (yellow oval). **b** OCT shows thumb‑shaped hemorrhagic PED (yellow arrows) and double layer sign (red arrow). **c** ICGA reveals PHS in nasal peripheral fundus (yellow oval). **d** OCT shows polypoidal lesion with sharp-peaked PED (yellow arrows). ICGA: indocyanine green angiography, OCT: optical coherence tomography, PED: pigment epithelial detachment, PHS: punctate hyperfluorescent spot

Two years and eight months later, the patient presented again with complaint of vision loss in her left eye. Corrected visual acuity of the left eye was 20/20, and fundus examination showed mild vitreous hemorrhage. Vitreous hemorrhage leading to retinal hemorrhage in the peripheral fundus was found. Vitreous surgery was performed with the aim to identify the cause of and to treat the vitreous hemorrhage. After vitrectomy, fundus examination revealed thick subretinal hemorrhage in the peripheral fundus, but no abnormality in the posterior pole (Fig. 2a). Choroid thickness at the macula was confirmed to be normal (203 μm) by OCT (Fig. 2b). In the temporal mid-peripheral region, late phase ICGA showed PHS with choroidal vascular hyperpermeability (Fig. 2c), and fluorescein angiography revealed CSC lesions with two inkblot leaks (Fig. 2d). At the inferior CSC lesion, pachychoroid (choroidal thickness 419 μm) was observed by OCT (Fig. 2e). In the superior CSC lesion, OCT revealed pachychoroid (choroidal thickness 439 μm) with pachyvessels and attenuated inner choroidal layers (Fig. 2f). In the peripheral fundus, ICGA depicted abnormal choroidal networks (Fig. 3a) and PHS (Fig. 3c), similar to the peripheral fundus findings in the right eye. Furthermore, OCT showed double layer sign and thumb‑shaped hemorrhagic PED (Fig. 3b). Furthermore, polypoidal lesions were confirmed by OCT (Fig. 3d). Based on the above findings, PEHCR with CSC and PHS were finally diagnosed in the left eye. Postoperative corrected visual acuity in the left eye was 20/20, and aflibercept intravitreal injection was performed for prophylactic purpose to prevent recurrence of vitreous hemorrhage. At 3 years after her first visit, her right eye vision remained at 20/20, and no PEHCR was found in the peripheral retina. Follow-up is ongoing.

## Discussion

We report for the first time a case diagnosed with PCV with PHS in one eye, and later PEHCR with CSC and PHS in the contralateral eye. This case suggests that PCV, PEHCR, and CSC may be linked pathologies involved in bilateral pachychoroid disease and choroidal vascular hyperpermeability. There is no clear criterion for pachychoroid, but Lim et al. [7] proposed choroidal thickness of 300 μm or more as indicating pachychoroid, as compared with less than 300 μm in normochoroid eye. In the present case, we diagnosed pachychoroid based on not only choroidal thickness, but also OCT findings of pachyvessels and attenuated inner choroidal layers, as well as ICGA finding of choroidal vascular hyperpermeability.

PHS are more frequently seen as a common choroidopathy of the posterior fundus in eyes with PCV and CSC than in eyes with AMD [8]. In the present case, PHS in the posterior fundus appeared to be located at the center of the choroidal vascular hyperpermeability on ICGA. Choroidal vascular hyperpermeability and PHS have been considered to be associated with pathologic choroidal vascular hyperpermeability conditions [9, 10], and choroidal vascular hyperpermeability has been speculated to contribute to the development of RPE protrusions that are observed as PHS on ICGA [9, 10]. In the present case, the PHS observed in the peripheral fundus were not associated with choroidal vascular hyperpermeability. Moreover, OCT of PHS revealed small sharp-peaked RPE elevation in peripheral fundus, but small RPE elevation in posterior fundus. Further investigation of this point is needed. In this case, the right eye was found to have PCV with PHS, and the left eye PEHCR with CSC and PHS. From these findings, PCV, PEHCR, and CSC may be linked pathologies manifesting pachychoroid and choroidal vascular hyperpermeability.

Diagnostic criteria for PEHCR have not been established. However the characteristics of PEHCR reported by Mantel et al. [5] may be useful for diagnosis: increased patient age (mean, 74 years; range, 60 to 88 years), female preponderance (55 %), pigment epithelium detachment (83 %), polyp-like structures in the choroid (69 %), abnormal choroidal vascular networks (50 %), subretinal pigment epithelium hemorrhage (44 %), lipid exudation (44 %), bilateral involvement (24 %), and sometimes extending to the macula. OCT revealed typical dome-shaped elevation of the pigment epithelium over the vascular polyps [5]. In the present case, the findings in the left eye met several of the above features; thus, PEHCR can be diagnosed.

PCV and PEHCR differ in lesion site: PCV in the macula and PEHCR in the peripheral fundus, but both can be regarded as mainly polypoidal lesion with pachychoroid [6]. Mantel et al. [5] reported that the abnormal choroidal vascular networks in PEHCR more closely resembled those observed in PCV than those seen in AMD. This observation, together with the number of clinical features that PEHCR share with PCV (hemorrhagic pigment epithelium detachment, lipid exudation, choroidal anomalous network, visible only on ICGA) suggest the possibility that PEHCR is a peripheral subtype of PCV [5]. Goldman et al. [11] and Goel [12] considered PEHRH as peripheral PCV, and Mashayekhi et al. [13] reported that PEHCR may be a variant of PCV.

A report has demonstrated that PEHCR shows female preponderance [14]. However, CSC occurs more frequently in males than females [15], and PCV shows ethnic variation and sex difference [16]. These pachychoroid spectrum diseases are phenotypes caused by the interaction between genotypes and environmental factors, which may account for the diverse ethnic variations and sex difference observed in this group of diseases [17]. *CFH* and *VIPR2* have been reported to be susceptibility loci in choroidal thickness and CSC [18]. Pachychoroid disease is thought to progress in the order of pachychoroid pigment epitheliopathy → CSC → pachychoroid neovasculopathy → PCV. In CSC, when the choriocapillaris is compressed by dilatation of the Haller layer of the choroid, ischemia may occur causing choroidal neovascularization, which leads to the development of pachychoroid neovasculopathy. In PCV, proliferation of choroidal capillaries under the RPE induces aneurysms similar to polyps at the tip. CSC is characterized by serous retinal detachment at the fovea. We believe that PEHCR may occur when choroidal vasodilation occurs due to an imbalance in choroidal circulation.

This case is valuable in demonstrating that PCV, PEHCR, and CSC may be linked pathologies of pachychoroid spectrum disease. The present report has limitations inherent of case reports. However, this report may stimulate further case reports or research on the association between PCV and PEHCR in bilateral pachychoroid disease.

## Conclusions

This is the first case report of PCV with PHS in one eye, and PEHCR with CSC and PHS in the contralateral eye. This case suggests that PCV, PEHCR, and CSC may be linked pathologies of pachychoroid spectrum disease.

## Data Availability

The clinical data used in the current study are available from the corresponding author on reasonable request.

## Abbreviations

CSC: Central serous chorioretinopathy
ICGA: Indocyanine green angiography
OCT: Optical coherence tomography
PCV: Polypoidal choroidal vasculopathy
PED: Pigment epithelial detachment
PEHCR: Peripheral exudative hemorrhagic choroidal retinopathy
RPE: Retinal pigmented epithelium
PHS: Punctate hyperfluorescent spot
